# Attenuation in Proinflammatory Factors and Reduction in Neuronal Cell Apoptosis and Cerebral Vasospasm by Minocycline during Early Phase after Subarachnoid Hemorrhage in the Rat

**DOI:** 10.1155/2021/5545727

**Published:** 2021-12-06

**Authors:** Chia-Li Chung, Hung-Pei Tsai, Yu-Hua Huang, Shu-Chuan Wu, Chee-Yin Chai, Aij-Lie Kwan

**Affiliations:** ^1^Graduate Institute of Medicine, College of Medicine, Kaohsiung Medical University, Kaohsiung, Taiwan; ^2^Department of Surgery, Kaohsiung Municipal Siaogang Hospital, Kaohsiung, Taiwan; ^3^Department of Neurosurgery, Kaohsiung Medical University Hospital, Kaohsiung, Taiwan; ^4^Department of Neurosurgery, Kaohsiung Chang Gung Memorial Hospital and Chang Gung University College of Medicine, Kaohsiung, Taiwan; ^5^Department of Pathology, Kaohsiung Medical University Hospital, Kaohsiung, Taiwan; ^6^Department of Pathology, College of Medicine, Kaohsiung Medical University, Kaohsiung, Taiwan; ^7^Department of Surgery, College of Medicine, Kaohsiung Medical University, Kaohsiung, Taiwan; ^8^Department of Neurosurgery, University of Virginia, Charlottesville, VA, USA

## Abstract

**Background:**

Subarachnoid hemorrhage (SAH) is an important subcategory of stroke due to its high mortality rate as well as severe complications such as neurological deficit. It has been suggested that cerebral inflammation is a major factor in advanced brain injury after SAH. Microglia and astrocytes are known supporting cells in the development and maintenance of inflammation in central nervous system. However, the role of microglia and astrocytes in the development of inflammation and neuronal cell apoptosis during the early phase after SAH has not been thoroughly investigated.

**Materials and Methods:**

Sprague-Dawley rats were divided into 4 groups (*n* = 6/group): sham group, animals subjected to SAH without treatment, SAH animals pretreated with the microglia inhibitor minocycline (50 mg/kg, ip), and SAH animals pretreated with the astrocyte inhibitor fluorocitrate (50 mg/kg, ip). SAH was induced by injecting autologous blood (1 ml/kg) into the cistern magna on day 0. Pretreatment with minocycline or fluorocitrate was given three days prior to the induction of SAH. Rats were sacrificed 6 hr after SAH, and their cerebral spinal fluids were used to measure protein levels of neuroinflammatory cytokines IL-1*β*, IL-6, and TNF-*α* by ELISA. In addition, the cerebral cortex was utilized to determine the levels of caspase-3 by western blot and to evaluate neuronal cell apoptosis by immunohistochemistry staining and detect microglia and astrocyte by immunofluorescence staining for Iba-1 and GFAP. In this study, all SAH animals were given an injection of autologous blood and SAH rats treated with minocycline or fluorocitrate received ip injections on day 1, 2, and 3 before inducing SAH. Neurological outcome was assessed by ambulation and placing/stepping reflex responses on day 7.

**Results:**

Immunofluorescence staining showed that SAH induced proliferation of microglia and astrocyte and minocycline inhibited the proliferation of both microglia and astrocyte. However, fluorocitrate inhibited only the proliferation of astrocyte. ELISA analysis showed that SAH upregulated TNF-*α* and IL-1*β*, but not IL-6 at 6 hr after SAH. Minocycline, but not fluorocitrate, attenuated the upregulation of TNF-*α* and IL-1*β*. Western blot analysis and immunohistochemistry staining showed that SAH induced neuronal cell apoptosis. Pretreatment with minocycline, but not fluorocitrate, decreased SAH-induced neuronal death and cerebral vasospasm. Furthermore, significant improvements in neurobehavioral outcome were seen in the minocycline treatment group, but not in animals treated with fluorocitrate.

**Conclusions:**

Microglia may play an important role to regulate neuronal cell apoptosis and cerebral vasospasm through inhibiting inflammation at an early phase after SAH in the rat.

## 1. Introduction

Subarachnoid hemorrhage (SAH) is a special type of hemorrhagic stroke, often characterized by spontaneous cerebral aneurysmal rupture and bleeding into the subarachnoid space [1]. Following SAH, 20-35% of patients die within a few days. Delayed cerebral ischemia (DCI) develops in the first few days up to several weeks after the initial bleeding and is the most important cause of poor outcome in SAH patients who survive in the first 24 hr [2]. Increasing reports suggest that cerebral inflammation is a major factor in advanced brain injury after SAH [3].

Inflammation processes are implicated in the development of ischemic stroke, and the levels of plasma inflammatory factors correlate with poor outcome [4, 5]. It has been shown that inflammatory factors such as TNF-*α*, IL-1*β*, and IL-6 in the cerebral spinal fluids (CSF) are associated with the development of ischemia and brain injury [6–8]. Furthermore, CSF concentrations of IL-6 have been found to be raised following SAH, especially in patients who later develop cerebral vasospasm.

Microglia and astrocytes support not only cells but also immune cells. Microglia is the resident central nervous system (CNS) immune cell. It plays an important role in neural development, repair, inflammation, infection, and neuropathic pain [9]. On the other hand, astrocytes are the best-characterized innate immune neuroglia. The main functions of astrocytes include buffering CNS potassium, removing and recycling potentially toxic glutamate, adjusting water balance, and modulating synaptic activity and blood flow. Both the microglia and astrocytes have been reported to release inflammatory factors to induce cell apoptosis, activate transcription factor, and hence yield positive feedback. However, little is known about the relationship between the microglia or astrocytes and SAH.

We hypotheses that microglia and astrocytes play important roles during the early phase brain injury after SAH. In the present study, rats were subjected to SAH and treated with minocycline (a microglia inhibitor) or fluorocitrate (an astrocyte inhibitor) to differentiate the involvement of microglia and astrocytes in this model of SAH. The effects of minocycline and fluorocitrate on the levels of inflammatory factors, neuronal cell apoptosis, and behavior in rats after SAH were evaluated.

## 2. Materials and Methods

### 2.1. Animals

Male Sprague-Dawley rats weighing 250–300 g were purchased from National Animal Center, Taiwan. All animal procedures were approved by the Kaohsiung Medical University animal research committee. After arriving at the Kaohsiung Medical University vivarium, the rats were acclimated for at least one week before being used in the experiment. They were housed in a room on a 12 hr light/dark cycle under controlled temperature of 22.1°C and relative humidity of 55%, and they were provided with normal chow and water ad libitum. Animals were randomly divided into four groups of six, and minocycline (50 mg/kg) and fluorocitrate (50 mg/kg) were injected intraperitoneally (ip) three days before the induction of SAH. At the beginning of the experiment, rats were anesthetized with Zoletil (50 mg/kg) ip, and fresh blood (1 ml/kg) was drawn from the central tail artery and injected into the cistern magna on day 0 to induce SAH.

### 2.2. Enzyme-Linked Immunosorbent Assay (ELISA)

Animals were sacrificed at 6 hr after SAH, and their 100 *μ*L cerebral spinal fluids (CSF) were collected to determine protein levels of neuroinflammatory cytokines IL-1*β*, IL-6, and TNF-*α* using eBioscience Platinum ELISA (eBioscience, Inc., CA, USA) according to the manufacturer's instructions.

### 2.3. Western Blot Analysis

For protein extraction, a single hemi-cord segment of cortex was homogenized in proteolysis buffer in the presence of protease inhibitors (Sigma, St. Louis, MO, USA) and incubated on ice for 10 min. Samples were centrifuged at 13,000 × rpm for 30 min at 4°C. Protein concentration in the supernatant was determined by the Bio-Rad DC Protein Assay Kit (Bio-Rad; 5000111). For Western blot analysis, 50 *μ*g proteins were separated by 12% sodium dodecyl-sulfate polyacrylamide gel electrophoresis (SDS-PAGE) and transferred onto PVDF membranes. After blocking in Tris-buffered saline containing 0.05% Tween-20 (TBST) and 5% nonfat milk for 1 hr at room temperature, the membranes were incubated overnight at 4°C with various primary antibodies [cleaved caspase-3 (1 : 200; Cell Signaling; #9661), *β*-actin (1 : 20000; Sigma; A5441)] directed against the protein of interest. After being washed twice, an appropriate HRP-conjugated secondary antibody (Millipore; P36599A) was applied for 1 hr at room temperature. Peroxidase activity was visualized using the ECL Western Blotting Detection kit (PerkinElmer; NEL122001EA) and X-ray films.

### 2.4. Tissue Processing

At the end of experiments, each animal is reanesthetized for perfusion and fixation. The thoracic cage is opened with canalling the left ventricle using a No. 16 catheter. After clamping the descending aorta and puncturing the right atrium, the brain is perfused with 180 mL of 2% paraformaldehyde and 100 mL of phosphate buffer (0.01 M) under 36°C and 100 mm Hg perfusion pressure. Gross inspection of harvested brains is performed to confirm the presence of subarachnoid blood clots over the BA (basilar artery), and the specimen is immersed in a fixative solution. The BAs are then separated from the brainstems, and the middle third of each vessel is dissected. The arterial segments are flat-embedded in paraffin, and BA cross-sections are cut into 3 *μ*m sections that are stained with hematoxylin and eosin stain for subsequent analysis.

### 2.5. Morphometric Assessment of BA

Three cross-sections from the middle-third BA from each animal are analyzed by a trained research staff who is blinded to the experimental groups. The thickness of BA is defined as the largest vertical distance between the inner surface of endothelium and the outer surface of adventitia. The arterial cross-sectional area is calculated using computer-based morphometric analysis (Image 1; Universal Imaging Corp., USA). The average area of BA cross-sections from each rat is calculated to obtain mean values for the degree of vasospasm at 48 h after SAH.

### 2.6. Immunofluorescence

After deparaffinization and rehydration, paraffin-embedded brain samples were treated by steam heating for antigen retrieval (30 min) using a DAKO antigen retrieval solution (DAKO, Carpenteria, CA). Slides were washed twice in TBS; the sections were incubated with mouse anti-GFAP (Sigma; G3893; USA) and rabbit anti-Iba1 (Proteintech; 10904-1-AP; Taiwan) antibodies for 16 hr at 4°C. Slides were again washed twice with TBS and subsequently incubated with Goat anti-Rabbit IgG (H+L)-FAM (Croyez; C04013; Taiwan) and Goat anti-Mouse IgG (H+L)-TAMRA (Croyez; C04012; Taiwan) antibody for 90 min at room temperature. After wards, the slides were washed twice with TBS. They were mounted using Fluoroshield™ with DAPI (Sigma; F6057; USA).

### 2.7. Immunohistochemistry

Paraffin-embedded brains from rats, after deparaffinization and rehydration, were treated by steam heating for 5 min to retrieve antigen using DAKO antigen retrieval solution (DAKO, Carpenteria, CA). They were then washed twice with Tris-buffered saline (TBS). Endogenous peroxidase was inhibited by immersing the slides in a 3% hydrogen peroxide solution for 5 min. Slides were again washed twice in TBS and incubated with primary antibody against cleaved caspase-3 (1 : 200; Cell Signaling; #9661) for 1 hr at room temperature. Slides were washed twice with TBS and subsequently incubated with biotinylated secondary antibody for 30 min. They were then washed twice with TBS and incubated with DAB (Dako; K5007) for 5 min, followed by washing twice with distilled water. Immediately after staining, slides were counterstained with hematoxylin for 1 min, rinsed for 1 s with distilled water, and dehydrated for 1-2 sec with 90–100% isopropanol. Finally, samples were immersed in xylene for 10 min, mounted using Permount (Fisher Scientific, Pittsburg, PA) and were examined using an inverted microscope (Olympus).

### 2.8. Neurological Assessment

Behavioral changes in the SAH rats were assessed by an investigator blinded to the experiment at day 7 after the injection of autologous blood. Assessment of ambulation (walking with lower extremities) and placing/stepping reflex (dragging the dorsum of hind paw over the edge of a surface) responses were used as an index of motor function according to a scoring system published previously [10]. The sum of scores from ambulation and placing/stepping reflex responses is referred to as motor deficit index (MDI).

### 2.9. Statistical Analysis

Western blot images were analyzed by Quantity One. All results were expressed as the mean ± SEM. Analysis of variance (ANOVA) was performed followed by the Tukey-Kramer post hoc test to determine statistical significance between two experimental groups. A *P* value of <0.05 was considered statistically significant.

## 3. Results

### 3.1. Minocycline Attenuated SAH-Induced Release of Proinflammatory Factors in CSF

**Figure 1:**
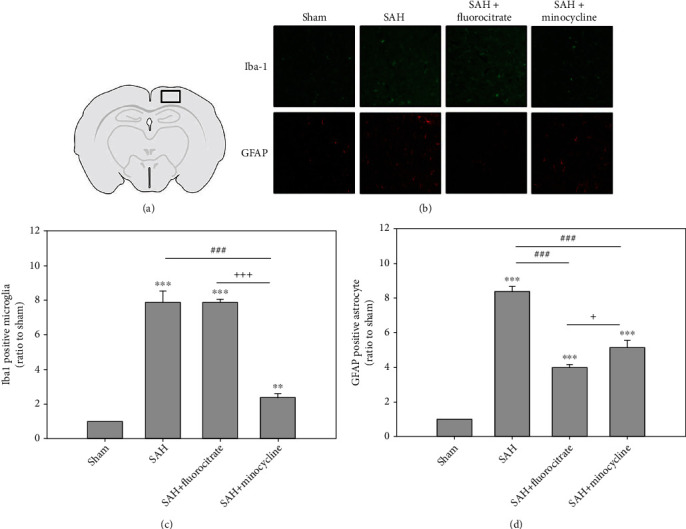
Proliferation of microglia and astrocyte in the cortex from a rat brain as determined by immunofluorescence staining for Iba-1 and GFAP, respectively. Representative micrographs of Iba-1 staining are shown. (a) The region for immunofluorescence staining of (b) sham, SAH, SAH+fluorocitrate (50 mg/kg), and SAH+minocycline (50 mg/kg). (c) The intensities of immunofluorescence staining for Iba-1 in the images were quantified relative to the levels of the sham group. (d) The intensities of immunofluorescence staining for GFAP in the images were quantified relative to the levels of the sham group. ^∗∗∗^*P* < 0.001 compared with sham group. ^##^*P* < 0.01 and ^###^*P* < 0.001 compared with the SAH+minocycline group. ^++^*P* < 0.01 and ^+++^*P* < 0.001 compared with the SAH+fluorocitrate group.

To demonstrate the effect of preretreatment with microglia and astrocyte minocycline and fluorocitrate, immunofluorescence satin for Iba-1 (microglia's marker) and GFAP (astrocyte's marker) was detected at 6 hr after SAH. In this early phase of SAH, Iba-1-positive microglia and GFAP-positive astrocyte in the SAH group were significantly higher than those in the sham group. Pretreatment with minocycline significantly both attenuated the Iba-1-positive microglia (*P* < 0.001) and GFAP-positive astrocyte (*P* = 0.001). However, pretreatment with fluorocitrate only significantly attenuated the GFAP positive astrocyte (*P* < 0.001). In addition, to demonstrate the relationship between proinflammatory factors and SAH, the levels of TNF-*α*, IL-1*β*, and IL-6 in CSF were examined using ELISA at 6 hr after SAH. In this early phase of SAH, protein expressions of TNF-*α* and IL-1*β* were significantly higher than that of the sham control, but IL-6 expression was not significantly different (Figure 1). Pretreatment with the microglia inhibitor minocycline at a dose of 50 mg/kg, ip, three days prior to the induction of SAH was shown to significantly decrease in TNF-*α* and IL-1*β* levels when compared with the untreated SAH group (Figures 1(a) and 1(b)). However, pretreatment with the astrocyte inhibitor fluorocitrate did not show significant differences when compared with the SAH group (Figure 1).

### 3.2. Minocycline Prevented SAH-Induced Neuronal Cell Apoptosis

**Figure 2:**
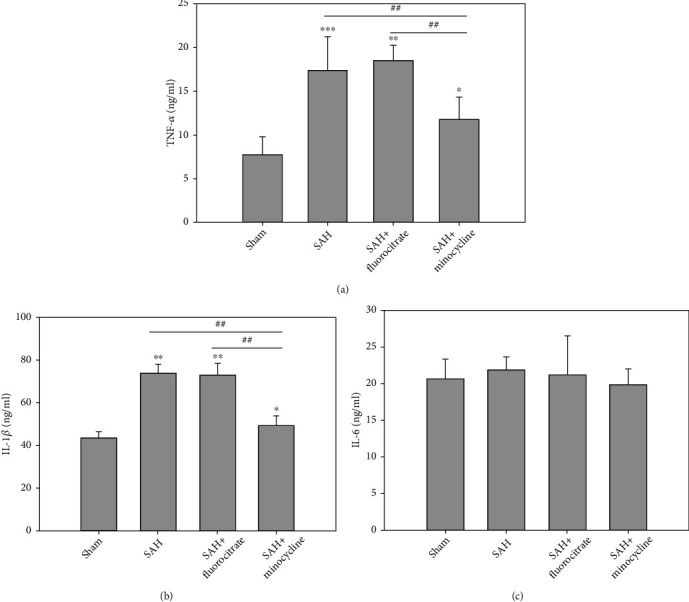
ELISA assay for TNF-*α*, IL-1*β*, and IL-6 in the CSF of rats subjected to SAH. Minocycline (50 mg/kg) or fluorocitrate (50 mg/kg) was injected intraperitoneally three days before surgery, and the CSF were collected at 6 hr after SAH was induced. (a) TNF-*α*, (b) IL-1*β*, and (c) IL-6. ^∗^*P* < 0.05 and ^∗∗^*P* < 0.01 compared with the sham group. ^#^*P* < 0.05 and ^##^*P* < 0.01 compared with the SAH+minocycline group.

**Figure 3:**
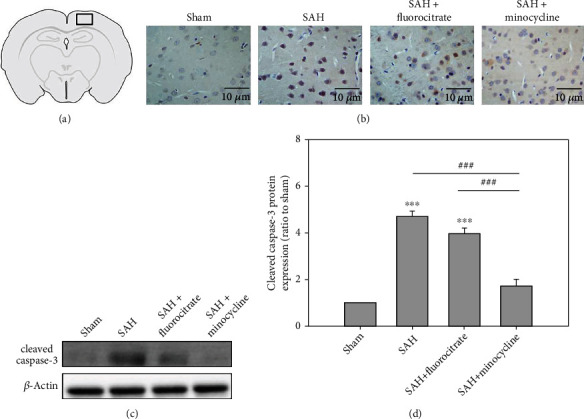
Immunohistochemistry staining of TUNEL assay and western blot analysis to show the effect of minocycline or fluorocitrate treatment on apoptosis in the cortex of rats subjected to SAH. (a) The region for immunohistochemistry staining of TUNEL assay. (b) Immunohistochemistry staining of TUNEL assay for minocycline (50 mg/kg) or fluorocitrate (50 mg/kg) was injected intraperitoneally three days prior to the induction of SAH, and the cortex tissues were collected at 6 hr postsurgery. (c) Western blot assay of cleaved caspase-3 for minocycline (50 mg/kg) or fluorocitrate (50 mg/kg) was injected intraperitoneally three days before surgery. (d) The cleaved caspase-3 expression levels were normalized sham group. ^∗∗∗^*P* < 0.001 compared with the sham group. ^###^*P* < 0.001 compared with the SAH+minocycline group.

Many studies reported upregulations of TNF-*α* and IL-1*β* in the early stage of apoptosis [11, 12]. The effect of SAH on neuronal cell apoptosis was evaluated using western blot analysis and immunohistochemistry staining for caspase-3 in the cortex. Western blot analysis showed that the level of cleavage caspase-3 in the SAH group was significantly higher than that of the sham group (Figure 2). Immunohistochemistry staining showed that the cleavage caspase-3 located in neuron cells, not in microglia or astrocyte (Figure 3(b)). These results supported that SAH induced neuronal cell apoptosis at early phase. Pretreatment with minocycline significantly decreased the levels of cleavage caspase-3 when compared with that of the SAH group (Figure 3(c)), whereas no significant effects were found in the fluorocitrate pretreatment group (Figure 3(d)). These results suggested that microglia, not astrocyte, induced neuronal cell apoptosis following inflammation.

### 3.3. The Mechanism of Minocycline following SAH

**Figure 4:**
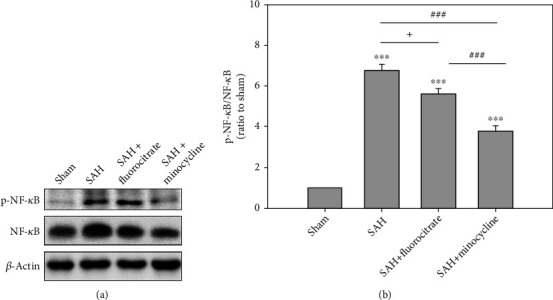
Western blot analysis to show the effect of minocycline or fluorocitrate treatment on the levels of p-NF-*κ*B/NF-*κ*B protein in the cortex of rats at 6 hr postsurgery. (a) Western blot assay of cleaved caspase-3 for minocycline (50 mg/kg) or fluorocitrate (50 mg/kg) was injected intraperitoneally three days before surgery. (b) The cleaved caspase-3 expression levels were normalized sham group. ^∗∗∗^*P* < 0.001 compared with the sham group. ^###^*P* < 0.001 compared with the SAH+minocycline group. ^+^*P* < 0.05 compared with the SAH+fluorocitrate group.

To detect the mechanism of minocycline following SAH, western blot analysis showed the phosphorylation of NF-*κ*B at 6 hr after SAH. In the early phase of SAH, the ratio of p-NF-*κ*B/NF-*κ*B in SAH was significantly higher than that of the sham group. Both pretreatments with minocycline and fluorocitrate were significantly decreased the ratio of p-NF-*κ*B/NF-*κ*B than the SAH group (Figure 4). However, the group from pretreatment with minocycline has a significantly lower ratio of p-NF-*κ*B/NF-*κ*B than the group from pretreatment with fluorocitrate (Figure 4).

### 3.4. Behavioral Assessment

Animals in the sham group had no neurological deficits and had a score of zero in both the ambulation and placing/stepping reflex responses. In contrast, the ambulation and placing/stepping responses scores in rats subjected to SAH were 1.30 ± 0.28 (mean ± SEM, *n* = 6) and 1.52 ± 0.37, respectively (both *P* < 0.05 compared to the sham group) (Table 1). The MDI value in the SAH group was 2.81 ± 0.30, also significantly different from that of the sham group. Pretreatment with fluorocitrate showed no significant improvement in MDI when compared with the SAH group. However, pretreatment with minocycline significant improved the ambulation and placing/stepping reflex scores to 0.84 ± 0.32 and 0.79 ± 0.11, respectively, when compared with the SAH group (*P* < 0.05 for both). The MDI value for the minocycline group (1.57 ± 0.22) also significantly decreased when compared with the SAH-only group (*P* < 0.05).

### 3.5. Morphological, Cross-Sectional Area, and Thickness Changes in BA

**Figure 5:**
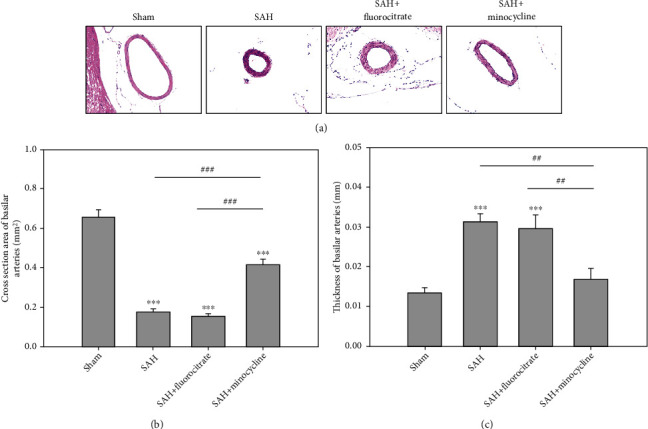
Representative micrographs of BA cross sections obtained from (a) sham, SAH, SAH+fluorocitrate (50 mg/kg), and SAH+minocycline (50 mg/kg). (b) Comparison of the BA cross-sectional area among sham, SAH, SAH+fluorocitrate (50 mg/kg), and SAH+minocycline (50 mg/kg). (c) Comparison of the thickness of BA among the same four groups. ^∗∗∗^*P* < 0.001 compared with the sham group. ^##^*P* < 0.01 and ^###^*P* < 0.001 compared with the SAH+minocycline group.

Upon microscopic examination, endothelial deformation, twisting of internal elastic laminae, and smooth muscle necrosis were seen in the BAs of rats subjected to sham, SAH, SAH+fluorocitrate, and SAH+minocycline (Figure 5(a)). The mean cross-sectional area of BA was 0.66 ± 0.036 mm^2^ in the sham group, 0.18 ± 0.013 mm^2^ in the SAH group, 0.15 ± 0.014 mm^2^ in the SAH+fluorocitrate group, and 0.42 ± 0.029 mm^2^ in the SAH+minocycline group (Figure 5(b)). The BA cross-sectional area in the SAH+minocycline group was reduced by 57.4% (*P* < 0.05) when compared with that in the SAH groups, respectively. In thickness of BA, there was no significant difference between the SAH (0.031 ± 0.002 mm) and SAH+fluorocitrate (0.030 ± 0.003 mm) groups (Figure 5(c)). A significant decrease in the thickness of BA was found in the SAH+minocycline group (0.017 ± 0.003 mm; *P* < 0.01 vs. SAH) (Figure 5(c)).

## 4. Discussion

Our study showed that minocycline could reduce the levels of proinflammatory factors and decrease neuronal cell apoptosis induced by SAH. Minocycline, known as a tetracycline antibiotic, has been considered anti-inflammatory, antiapoptotic, and antioxidative, resulting from its ability to downregulate matrix metalloproteinase- (MMP-) 9, phospholipase A2, phosphorylation of NF-*κ*B, and interleukin- (IL-) 1*β* converting enzyme [13–18]. It was also found to reduce hippocampal damage 24 hr after SAH, and its neuroprotective potential has been reported for a number of different neurological disorders, neurodegenerative diseases, and traumatic brain injury [19]. SAH induced oxidative stress and inflammation through phosphorylation of NF-*κ*B [20, 21]. Minocycline is also a potent iron chelator forming iron complexes to attenuate the inflammatory response by reducing neurotoxic stimulus and ameliorate the toxic effects of free subarachnoid hemoglobin, which is likely to be a trigger of inflammation after SAH [13, 22]. In our result, minocycline injection significantly attenuated the protein ratio of p-NF-*κ*B/NF-*κ*B at 6 hr after SAH. Therefore, minocycline attenuated the inflammation through inhibiting p-NF-*κ*B in the early phase of SAH.

Inflammation and cytokines may participate in the pathology of blood brain barrier (BBB) disruption and brain edema, which are characteristic features for both clinical and experimental SAH [23, 24]. A variety of inflammatory cytokines, including IL-1*β*, IL-6, and TNF-*α*, are strongly associated with brain injury in the rat [25]. Inhibition of IL-1*β* has been shown to attenuate early brain injury (EBI) and improve BBB function after SAH [26]. The present study showed that minocycline decreased the SAH-induced production of IL-1*β* (Figure 1). The precise mechanism by which inhibition of IL-1*β* production leads to a significant improvement in the ambulation and placing/stepping responses seen with minocycline treatment in SAH rats has not been fully elucidated at the present.

In addition to the anti-inflammatory effects, minocycline has also been shown to have antiapoptotic action in various experimental animal models [19, 27, 28]. It is possible that minocycline reduces the expression of caspase-3 and MMPs and activates microglia [29, 30]. Both the anti-inflammatory and antiapoptotic effects of minocycline may also involve the reduction of reactive oxygen species (ROS) levels [31]. In the present study, we also found minocycline suppressed the expression caspase-3 in rats after SAH (Figure 2). Whether or not this antiapoptotic effect of minocycline was resulted from inhibition of ROS remains to be investigated. In this study, immunofluorescence satin showed that minocycline injection decreased both Iba-1- and GFAP-positive cells, but fluorocitrate injection decreased only GFAP-positive cells. ELISA assay showed the level TNF-*α* and IL-1*β* in the SAH+minocycline group were significantly lower than the SAH group, but those in the SAH+fluorocitrate group were not different than those in the SAH group. These data supported minocycline, a microglia inhibitor, attenuated inflammation and neuron apoptosis in the early phase of SAH. Therefore, microglia may upregulate astrocyte to release inflammatory factors to induce neuron apoptosis in the early phase of SAH.

Astrocytes are essential components of neurovascular unit and play a role in the dysfunctions following SAH [32, 33]. It has been suggested that delayed astrocytic apoptosis might be involved in the development of delayed brain injury after SAH [1]. In contrast to the effects observed with minocycline treatment, inhibition of the astrocytes by fluorocitrate did not reduce the levels of proinflammatory factors nor did it decrease neuron cell apoptosis induced by SAH in the present study. Differences in animal SAH models might account for this discrepancy.

There are different phases of SAH-induced effects. The present study focused on the early phase effects in rats subjected to SAH. Consistent with our findings, microglia-dependent neuronal apoptosis was found in the early phase following SAH [28]. Another publication showed microglia seemed to be both necessary and sufficient to cause vasospasm in both the early and late phases of SAH [34]. These studies supported the involvement of microglia in the early phase of SAH. However, the role that microglia may play in the late phase of SAH still needs to be clarified.

## 5. Conclusions

In our SAH model, we found inhibition of microglia by minocycline decreased the proinflammatory factors release and reduced neuronal cell apoptosis and cerebral vasospasm. Multiple treatment with minocycline also improved the outcome in behavioral assessments. However, these effects were not seen after in inhibition of astrocytes by fluorocitrate. We suggested the microglia inhibition may lead to neuroprotective effect and attenuate cerebral vasospasm, and regulation of activated microglia will be of therapeutic benefit in the early phase of aneurysmal SAH in the future.

## Figures and Tables

**Table 1:** Behavioral assessment.

Treatment	Ambulation	Placing/stepping reflex	MDI
Sham	0	0	0
SAH	1.30 ± 0.28	1.52 ± 0.37	2.81 ± 0.30
SAH+fluorocitrate	1.5 ± 0.18	1.33 ± 0.17	2.79 ± 0.39
SAH+minocycline	0.84 ± 0.32	0.79 ± 0.11^∗^	1.57 ± 0.22^∗^

Rats were anesthetized with a mixture of KetaVed and xylazine intraperitoneally (ip), and fresh blood (1 ml/kg) was drawn from the central tail artery and injected into the cistern magna on day 0 to induce SAH. All SAH animals were given a second injection of autologous blood on day 5. Treatments with minocycline (50 mg/kg, ip) or fluorocitrate (50 mg/kg, ip) were given on day 1, 2, and 3 before inducing SAH. Neurological outcome was assessed by ambulation and placing/stepping reflex responses on day 7. MDI (motor deficit index) which is the sum of ambulation (walking with lower extremities) and placing/stepping reflex (dragging the dorsum of hind paw over the edge of a surface) scores.

## Data Availability

The all data including picture, quantitative chart, and table of neurobehavior used to support the findings of this study are included within the article.

## References

[1] Sabri M., Kawashima A., Ai J., Macdonald R. L. (2008). Neuronal and astrocytic apoptosis after subarachnoid hemorrhage: a possible cause for poor prognosis. *Brain research*.

[2] van Gijn J., Kerr R. S., Rinkel G. J. (2007). Subarachnoid haemorrhage. *Lancet*.

[3] Zhang B. F., Song J. N., Ma X. D. (2015). Etanercept alleviates early brain injury following experimental subarachnoid hemorrhage and the possible role of tumor necrosis factor-*α* and c-Jun N-terminal kinase pathway. *Neurochemical Research*.

[4] Vila N., Castillo J., Davalos A., Chamorro A. (2000). Proinflammatory cytokines and early neurological worsening in ischemic stroke. *Stroke*.

[5] Smith C. J., Emsley H. C., Gavin C. M. (2004). Peak plasma interleukin-6 and other peripheral markers of inflammation in the first week of ischaemic stroke correlate with brain infarct volume, stroke severity and long-term outcome. *BMC Neurology*.

[6] Aly H., Khashaba M. T., El-Ayouty M., El-Sayed O., Hasanein B. M. (2006). IL-1*β*, IL-6 and TNF-*α* and outcomes of neonatal hypoxic ischemic encephalopathy. *Brain & Development*.

[7] Sumanovic-Glamuzina D., Culo F., Culo M. I., Konjevoda P., Jerkovic-Raguz M. (2017). A comparison of blood and cerebrospinal fluid cytokines (IL-1beta, IL-6, IL-18, TNF-alpha) in neonates with perinatal hypoxia. *Bosnian Journal of Basic Medical Sciences*.

[8] Hendryk S., Jarzab B., Josko J. (2004). Increase of the IL-1 beta and IL-6 levels in CSF in patients with vasospasm following aneurysmal SAH. *Neuro Endocrinology Letters*.

[9] Olson J. K. (2010). Immune response by microglia in the spinal cord. *Annals of the New York Academy of Sciences*.

[10] Huang Y. H., Chung C. L., Tsai H. P. (2017). Hyperglycemia aggravates cerebral vasospasm after subarachnoid hemorrhage in a rat model. *Neurosurgery*.

[11] Tu X. K., Chen Q., Chen S., Huang B., Ren B. G., Shi S. S. (2021). GLP-1R agonist liraglutide attenuates inflammatory reaction and neuronal apoptosis and reduces early brain injury after subarachnoid hemorrhage in rats. *Inflammation*.

[12] Shao A., Wu H., Hong Y. (2016). Hydrogen-rich saline attenuated subarachnoid hemorrhage-induced early brain injury in rats by suppressing inflammatory response: possible involvement of NF-*κ*B pathway and NLRP3 inflammasome. *Molecular Neurobiology*.

[13] Stirling D. P., Koochesfahani K. M., Steeves J. D., Tetzlaff W. (2005). Minocycline as a neuroprotective agent. *The Neuroscientist*.

[14] Guo Z. D., Wu H. T., Sun X. C., Zhang X. D., Zhang J. H. (2011). Protection of minocycline on early brain injury after subarachnoid hemorrhage in rats. *Acta Neurochirurgica. Supplement*.

[15] Dalm D., Palm G. J., Aleksandrov A., Simonson T., Hinrichs W. (2010). Nonantibiotic properties of tetracyclines: structural basis for inhibition of secretory phospholipase A_2_. *Journal of Molecular Biology*.

[16] Koistinaho M., Malm T. M., Kettunen M. I. (2005). Minocycline protects against permanent cerebral ischemia in wild type but not in matrix metalloprotease-9-deficient mice. *Journal of Cerebral Blood Flow and Metabolism*.

[17] Cai Z., Zhao Y., Yao S., Bin Z. B. (2011). Increases in *β*-amyloid protein in the hippocampus caused by diabetic metabolic disorder are blocked by minocycline through inhibition of NF-*κ*B pathway activation. *Pharmacological Reports*.

[18] Yi Q., Tan F. H., Tan J. A. (2019). Minocycline protects against myocardial ischemia/reperfusion injury in rats by upregulating MCPIP1 to inhibit NF-*κ*B activation. *Acta Pharmacologica Sinica*.

[19] Matsukawa N., Yasuhara T., Hara K. (2009). Therapeutic targets and limits of minocycline neuroprotection in experimental ischemic stroke. *BMC Neuroscience*.

[20] Sun X., Ji C., Hu T., Wang Z., Chen G. (2013). Tamoxifen as an effective neuroprotectant against early brain injury and learning deficits induced by subarachnoid hemorrhage: possible involvement of inflammatory signaling. *Journal of Neuroinflammation*.

[21] Suzuki H. (2019). Inflammation: a good research target to improve outcomes of poor-grade subarachnoid hemorrhage. *Translational Stroke Research*.

[22] Bahrami F., Morris D. L., Pourgholami M. H. (2012). Tetracyclines: drugs with huge therapeutic potential. *Mini Reviews in Medicinal Chemistry*.

[23] Zetterling M., Hallberg L., Hillered L., Karlsson T., Enblad P., Ronne E. E. (2010). Brain energy metabolism in patients with spontaneous subarachnoid hemorrhage and global cerebral edema. *Neurosurgery*.

[24] Altay O., Suzuki H., Hasegawa Y. (2012). Isoflurane attenuates blood-brain barrier disruption in ipsilateral hemisphere after subarachnoid hemorrhage in mice. *Stroke*.

[25] Maddahi A., Povlsen G. K., Edvinsson L. (2012). Regulation of enhanced cerebrovascular expression of proinflammatory mediators in experimental subarachnoid hemorrhage via the mitogen-activated protein kinase kinase/extracellular signal-regulated kinase pathway. *Journal of Neuroinflammation*.

[26] Sozen T., Tsuchiyama R., Hasegawa Y. (2009). Role of interleukin-1beta in early brain injury after subarachnoid hemorrhage in mice. *Stroke*.

[27] Sanchez Mejia R. O., Ona V. O., Li M., Friedlander R. M. (2001). Minocycline reduces traumatic brain injury-mediated caspase-1 activation, tissue damage, and neurological dysfunction. *Neurosurgery*.

[28] Choi Y., Kim H. S., Shin K. Y. (2007). Minocycline attenuates neuronal cell death and improves cognitive impairment in Alzheimer's disease models. *Neuropsychopharmacology*.

[29] Krady J. K., Basu A., Allen C. M. (2005). Minocycline reduces proinflammatory cytokine expression, microglial activation, and caspase-3 activation in a rodent model of diabetic retinopathy. *Diabetes*.

[30] Scholz R., Sobotka M., Caramoy A., Stempfl T., Moehle C., Langmann T. (2015). Minocycline counter-regulates pro-inflammatory microglia responses in the retina and protects from degeneration. *Journal of Neuroinflammation*.

[31] Elewa H. F., Hilali H., Hess D. C., Machado L. S., Fagan S. C. (2006). Minocycline for short-term neuroprotection. *Pharmacotherapy*.

[32] Harris J. J., Jolivet R., Attwell D. (2012). Synaptic energy use and supply. *Neuron*.

[33] Mishra A., Reynolds J. P., Chen Y., Gourine A. V., Rusakov D. A., Attwell D. (2016). Astrocytes mediate neurovascular signaling to capillary pericytes but not to arterioles. *Nature Neuroscience*.

[34] Hanafy K. A. (2013). The role of microglia and the TLR4 pathway in neuronal apoptosis and vasospasm after subarachnoid hemorrhage. *Journal of Neuroinflammation*.

