# Expression of Concern on “Attenuation in Proinflammatory Factors and Reduction in Neuronal Cell Apoptosis and Cerebral Vasospasm by Minocycline during Early Phase after Subarachnoid Hemorrhage in the Rat”

**DOI:** 10.1155/bmri/9767649

**Published:** 2026-04-08

**Authors:** BioMed Research International

EXPRESSION OF CONCERN: C.‐L. Chung, H.‐P. Tsai, Y.‐H. Huang, S.‐C. Wu, C.‐Y. Chai, and A.‐L. Kwan, “Attenuation in Proinflammatory Factors and Reduction in Neuronal Cell Apoptosis and Cerebral Vasospasm by Minocycline during Early Phase after Subarachnoid Hemorrhage in the Rat,” *BioMed Research International* 2021, 5545727. https://doi.org/10.1155/2021/5545727.

This Expression of Concern is for the above article, published online on 06 December 2021 in Wiley Online Library (wileyonlinelibrary.com), and has been issued by agreement between the journal’s Editor‐in‐Chief, Yoel A. Klug; and John Wiley & Sons Ltd. A third party reported on PubPeer [1] that the SAH data included in Table 1 was identical to the SAH data reported in an earlier article by many of the same authors [Chang et al. 2010 (10.1007/s00701-010-0652-3)]. As the articles reported on studies with different experimental conditions, the same data being reported twice is considered highly improbable.

The authors responded to an inquiry by the publisher, but they did not provide original data to resolve the concerns.

The Expression of Concern has been agreed to because the evidence of identical data between these two publications casts doubt on the originality of the results presented in the article. The authors have been informed of the Expression of Concern.
